# The complete mitochondrial genome of *Neuroctenus taiwanicus* (Hemiptera: Aradidae)

**DOI:** 10.1080/23802359.2024.2335986

**Published:** 2024-05-07

**Authors:** Zhancheng Jia, Rina Su, Xiaoshuan Bai

**Affiliations:** a Institute of Life Science and Technology, Inner Mongolia Normal University, Hohhot, China; b College of Plant Protection, Northwest A&F University, Shaanxi, Xianyang, China

**Keywords:** Mitochondrial genome, neuroctenus, neuroctenus taiwanicus

## Abstract

*Neuroctenus taiwanicus* Kormilev, 1955 is a flat-bodied and enigmatic bug that was first discovered on the island of Taiwan, China. In this study, the whole mitochondrial genome of *N. taiwanicus* was sequenced and annotated for the first time, and its genomic data were uploaded to Genbank feedback number OR675057. The mitochondrial genome of *N. taiwanicus* is 15,340 bp in length, a typical circular DNA encoding 37 genes and a control region with 68.4% A + T content. The phylogeny reveals the taxonomic status of *N. taiwanicus*, which is most closely related to *N. yunnanensis*, and demonstrates the sister relationship among *Neuroctenus*, *Mezira*, and *Brachyrhynchus*. In addition, the results also confirm that Aradinae and Calisiinae are the earliest branching and more primitive in the family Aradidae, which is consistent with the analysis of the traditional classification.

## Introduction

**Figure 1.**
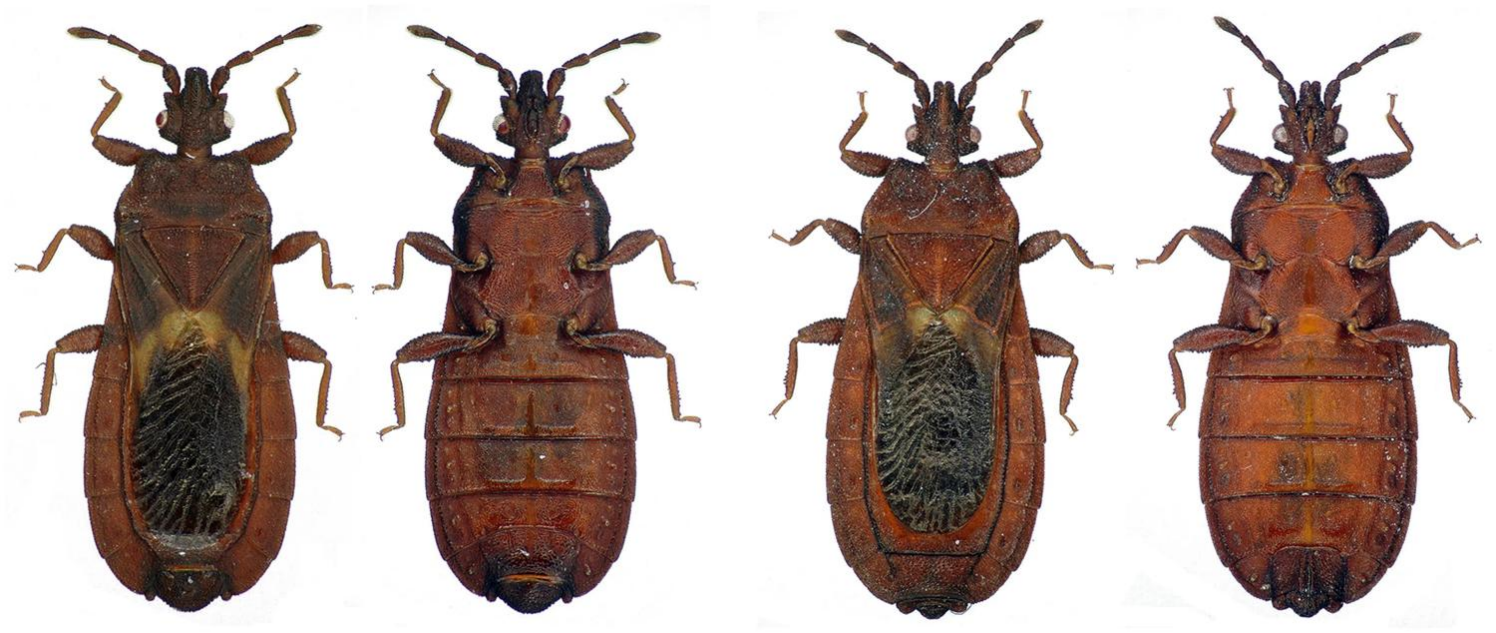
Species reference image of *Neuroctenus taiwanicus*. A, B-male; C, D-female; A, C-dorsal view; B, D-ventral view. (The photos of *N. taiwanicus* were taken by Xiaoshuan Bai in the Animal Lab College of Life Science and Technology, Inner Mongolia Normal University, China.)

*Neuroctenus taiwanicus* Kormilev 1955 (Hemiptera: Aradidae) is a member of Mezirinae, which was first discovered in Taiwan Island, China (Kormilev 1955). It has a very flat body, feeds on mycelium, and usually lives under the bark of fallen trees, occasionally hiding in crevices of the bark due to its protective coloring, which makes it difficult to detect; thus, they are full of mystery (Zha et al. 2023; Zhu et al. 2023). It is clearly distinguished from other insects by its ferrugineous body and yellowish basal membrane (Figure 1). It is similar to *N. yunnanensis*, but they are slightly different, and their identification has become a taxonomic problem. At the same time, they are excellent for studying phylogenies based on the fact that they are adaptable but rarely fly (Monteith, 1997). Currently, there are fewer molecular research efforts in Aradidae insects compared to other families, and even in *Neuroctenus*, a larger genus in Aradidae, only *N. parus* (Hua et al. 2008), *N. yunnanensis*, and *N. sp.* (Ye et al. 2022) have completed mitochondrial whole genome sequencing. In this study, the whole mitochondrial genome of *N. taiwanicus* was sequenced and annotated for the first time, and its phylogenetic relationships were investigated and discussed, which provides valuable genetic information for further studies on flat bugs.

## Materials and methods

The specimen of *Neuroctenus taiwanicus* used in this experiment was found in Dawei Mountain (22.9500 N, 103.5356E), Pingbian Miao Autonomous County, Yunnan Province, on October 10, 2019. We uncovered these flat bugs on a fallen tree on the mountain, picked them up with tweezers, soaked them in 95% alcohol, and brought them back to the laboratory to be identified by Professor Xiaoshan Bai from Inner Mongolia Normal University. The test specimen, voucher number DWSnt-a, is currently immersed in 95% ethanol and stored at −20 °C in the Zoological Museum of Inner Mongolia Normal University (http://bio.imnu.edu.cn/, Bai XS, baixs2007@aliyun.com).

Total DNA from the head, thorax and feet of *N. taiwanicus* was extracted using TIANamp genomic DNA kit of Tiangen Biotechnology Co., LTD., and sent to BerryGenomics (Beijing, China) for sequencing using the Illumina NovaSeq 2500 platform. De novo assemblies were conducted with Geneious Prime 2023 (Kearse et al. 2012). Annotations were performed using the MITOS online server (http://mitos.bioinf.uni-leipzig.de/index.py)(Bernt et al. 2013). We downloaded the whole mitochondrial genomes of nine flat bugs from NCBI (http://www.ncbi.nlm.nih.gov/) and used homology matching to manually correct the results of the 13 PCGs using MEGA X software (Kumar et al. 2018) and to obtain locus information and base content. Final mitochondrial visualization by CGView web server (https://cgview.ca/)(Grant and Stothard 2008). The annotated mitochondrial genome was uploaded to the NCBI database to obtain the accession number OR675057.

To reveal the phylogenetic position of *N. taiwanicus*, we downloaded nucleotide sequences of the 13 PCGs from 16 species of insects from the NCBI. A total of 17 species, including *Populicerus populi* (Hemiptera: Cicadellidae) (Wang et al. 2018) as an outgroup and *N. taiwanicus* in this experiment, were used to construct a phylogenetic tree based on Maximum-Likelihood method (ML) with Phylosuite v1.2.3 software (Zhang et al. 2020), and the model was selected as GTR + I + G + X. Finally, the phylogenetic tree was trimmed and beautified using the online website TVBOT (https://www.chiplot.online/tvbot.html) (Xie et al. 2023).

## Results

**Figure 2.**
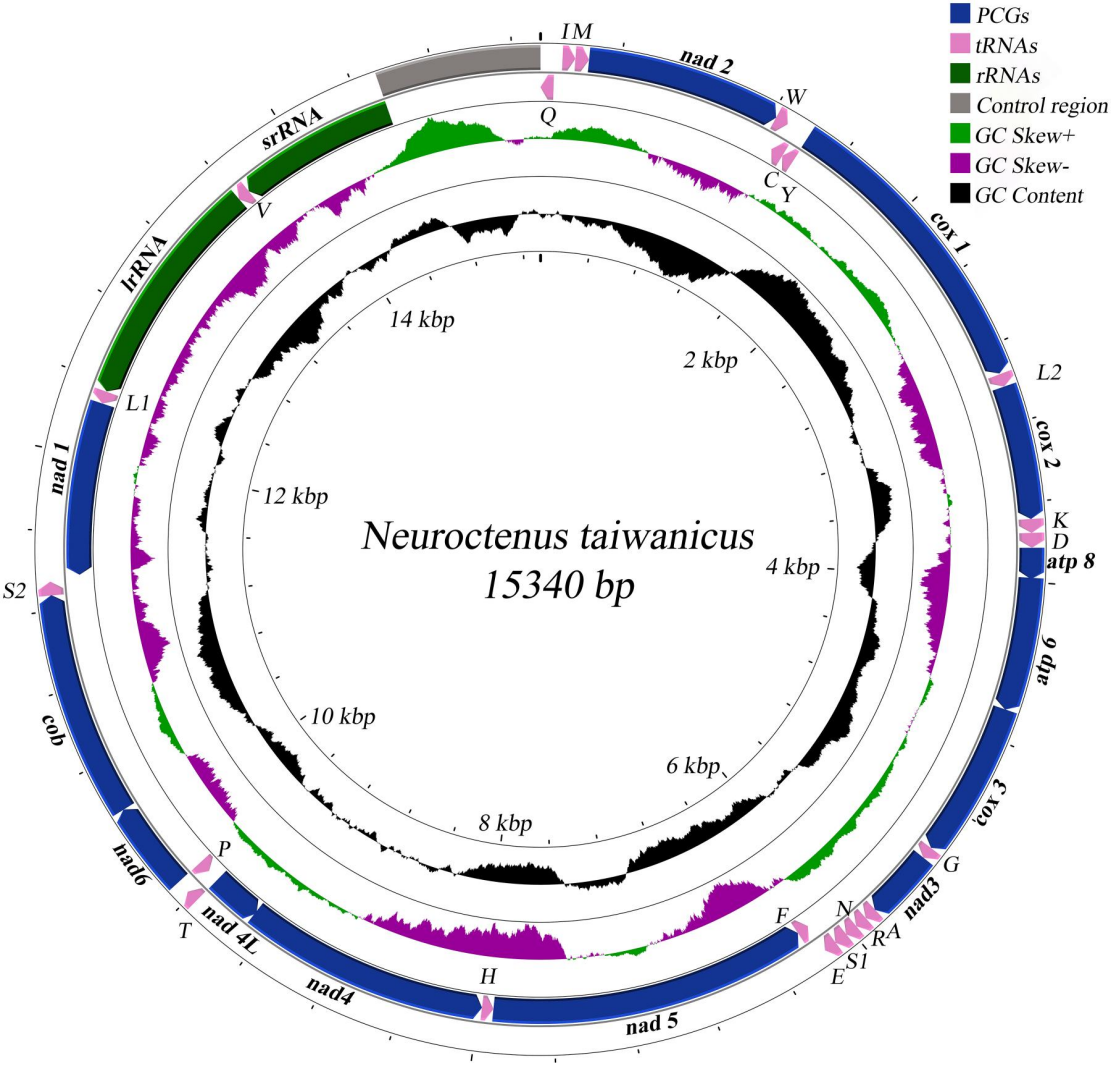
Mitochondrial genome map of *Neuroctenus taiwanicus.*

As in most insects, the molecular structure of *N. taiwanicus* mitochondrial DNA is circular, with a total length of 13,450 bp, containing 37 coding genes (13 PCGs, 22 tRNA genes, and 2 rRNA genes) and a control region (Figure 2). Of these, 23 genes are encoded on the Heavy strand (H-strand), and the remaining genes are located on the Light strand (L-strand). Like Aradidae insects, they do not have the same gene order as the insect ancestor (Song et al. 2012), and a rearrangement of tRNAs was found, with the positions of trnQ and trnI displaced (Song et al. 2016). The base composition was 41% A, 27.4% T, 19% C, and 12.6% G, showing a clear AT bias.

The total length of the 13 protein-coding genes of *N. taiwanicus* was 10936 bp, encoding 3635 amino acids. Ten PCGs used ATN (N=A, T, C, G) as the start codon, and the nad6 gene started with a rare ATC; the other three genes (cox1, cox2, and nad1) used TTG as the start codon. All ten PCGs have either TAA or TAG as a stop codon, and the other three genes (nad2, cox2, nad5, and nad4) end with a T residue.

**Figure 3.**
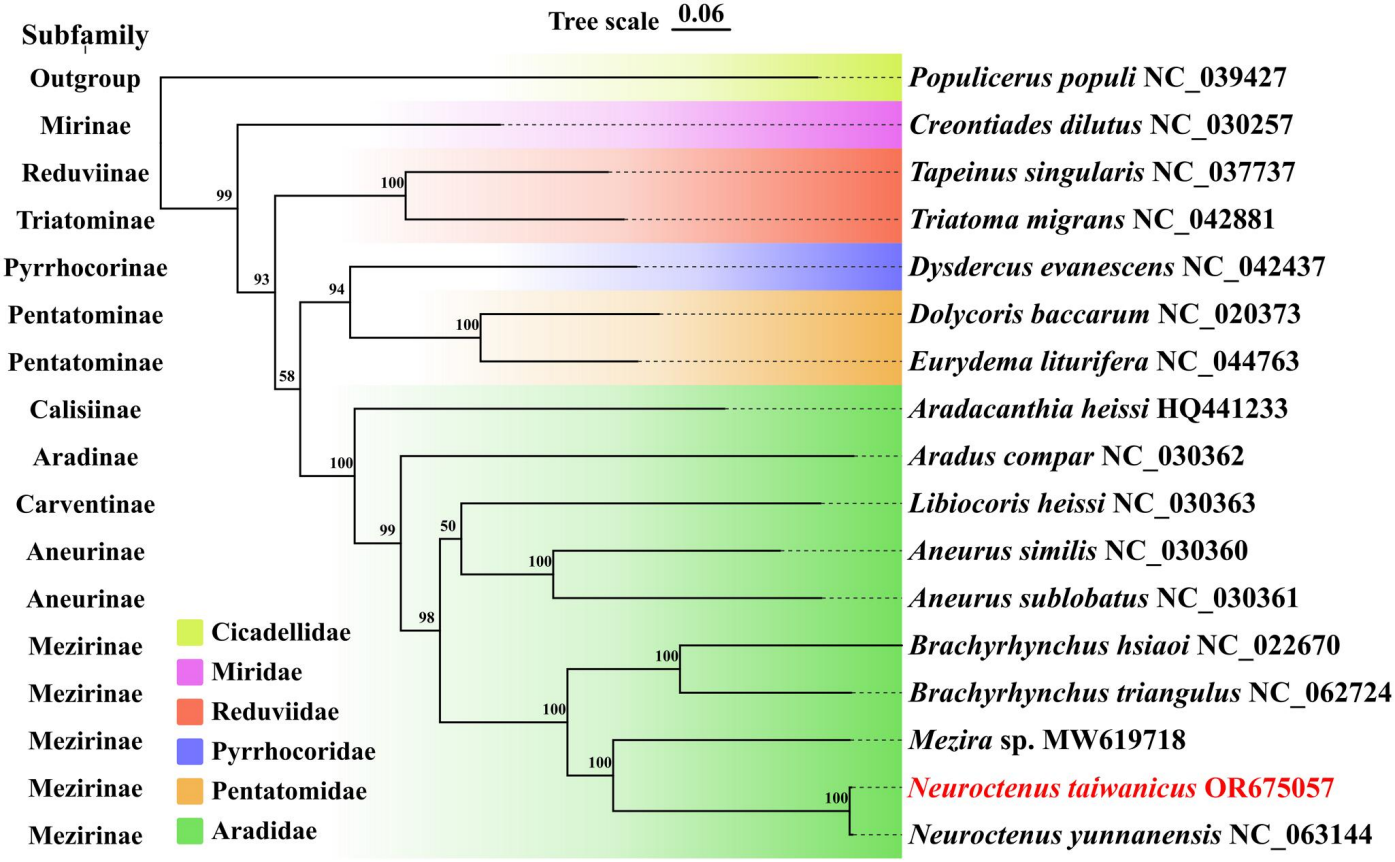
Phylogenetic tree obtained from Maximum-Likelihood (ML) analysis based on PCGs of 17 species. The best-fit evolutionary model is GTR + I + G + X. The complete mitochondrial sequences and accession ID were used as follows: *Populicerus populi* NC039427 (Wang et al. 2018); *Creontiades dilutus* NC030257 (Hereward 2016); *Tapeinus singularis* NC037737; *Triatoma migrans* NC042881 (Zhao et al. 2019); *Dysdercus evanescens* NC042437 (Liu et al. 2019); *Dolycoris baccarum* NC020373 (Zhang et al. 2013); *Eurydema liturifera* NC044763; *Aradacanthia heissi* HQ441233 (Shi et al. 2012); Aradus compar NC030362, *Libiocoris heissi* NC030363, *Aneurus similis* NC030360, *Aneurus sublobatus* NC030361 (Song et al. 2016); *Brachyrhynchus hsiaoi* NC022670 (Li et al. 2016); *Brachyrhynchus triangulus* NC062724 (Zhu et al. 2023); *Mezira sp.*, *Neuroctenus yunnanensis* (Ye et al. 2022).

We constructed a phylogenetic tree based on the ML method with Cicadellidae as the outgroup (Figure 3), and the results showed that *N. taiwanicus* and *N. yunnanensis* are the closest relatives, and *Neuroctenus* is the sister group to *Mezira* and *Brachyrhynchus*, which are all in the Mezirinae. At the same time, it also reveals that *Aradacanthia heissi* and *Aradus compar* are more primitive taxa. In addition, the close affinity between Aradidae and Pentatomidae was confirmed (Song et al. 2016). The results are in agreement with those of traditional taxonomic analyses.

## Discussion and conclusions

Flat bugs are typical phylogenetic model taxon due to their weak migratory capacity. However, the fact that the flatworm lives under the bark of trees or carries a protective coloration makes it infrequently appear in people’s eyes, so its taxonomic and molecular studies are not sufficient. In this paper, we show for the first time that the mitochondrial genome structure of *Neuroctenus taiwanicus* is a typical circular DNA with 37 coding genes, and some tRNA genes are rearranged. Interestingly, its mitochondrial genome are similar to *N.yunnanensis*, although there are some differences in antennae and genital segments. Phylogenetic trees based on 13 PCGs of the mitochondrial genome also support a close relationship between *N. teraiwanicus* and *N. yunnanensis*, possibly due to the short differentiation time between them. In conclusion, the taxonomic status of *N. teraiwanicus* is consistent with traditional taxonomic studies. This provides data support for unraveling the mystery of Aradidae and lays a good foundation for subsequent genetic work.

## Supplementary Material

Supplemental Material

Supplemental Material

Supplemental Material

## Data Availability

The genome sequence data annotation results of this study are publicly available in GenBank under accession number OR675057. The associated BioProject, SRA and Bio-Sample numbers are PRJNA1031840, SRR26589680 and SAMN37973943, respectively.
