# Supplementary figures and images for: The complete mitochondrial genome of Neuroctenus taiwanicus (Hemiptera: Aradidae)
Source: Mitochondrial DNA B Resour. 2024 May 7;9(5):597–600. doi: 10.1080/23802359.2024.2335986 (PMC11078071; doi:10.1080/23802359.2024.2335986)

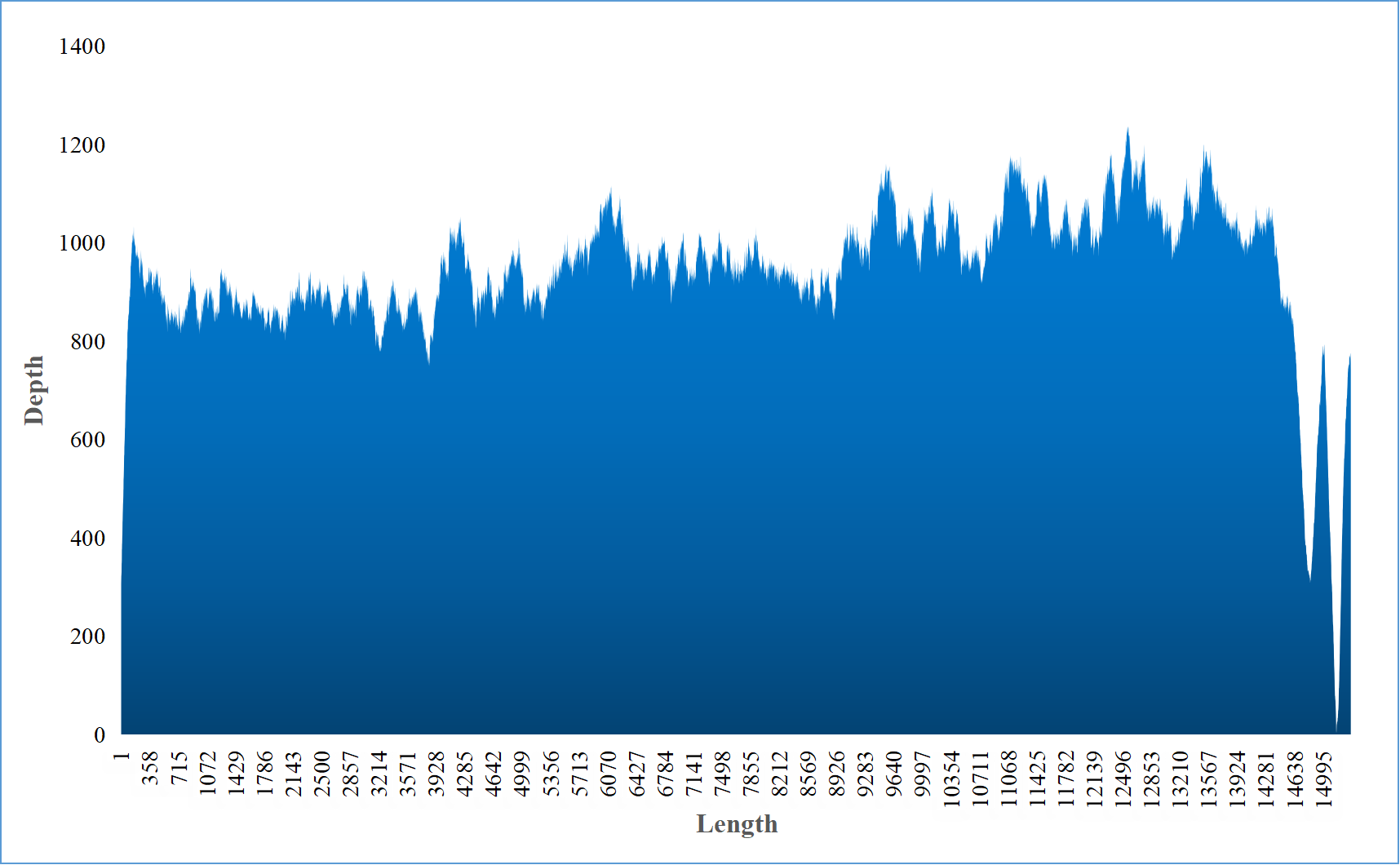

Supplement: Supplemental Material [file TMDN_A_2335986_SM4098.png]

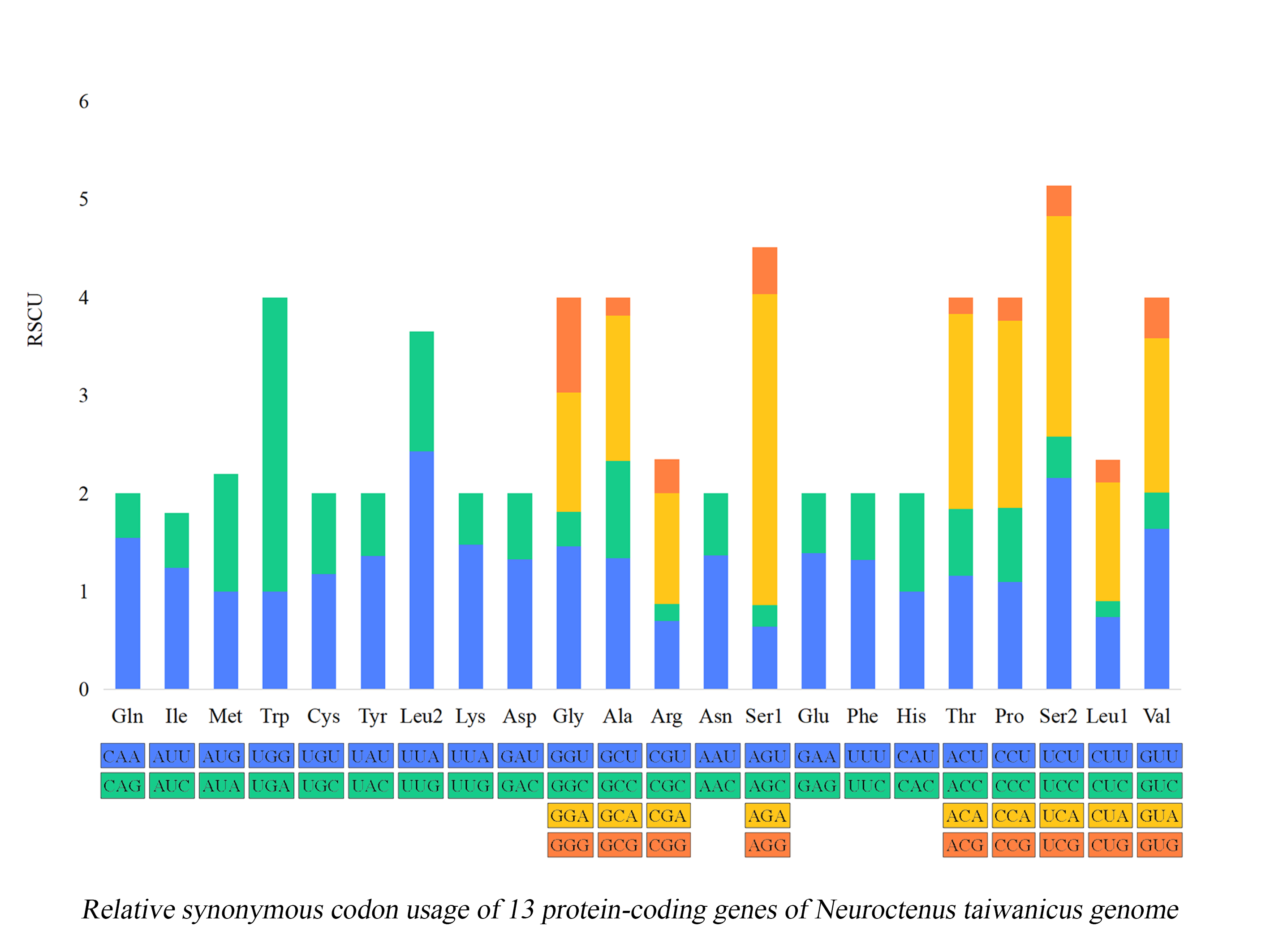

Supplement: Supplemental Material [file TMDN_A_2335986_SM3849.png]

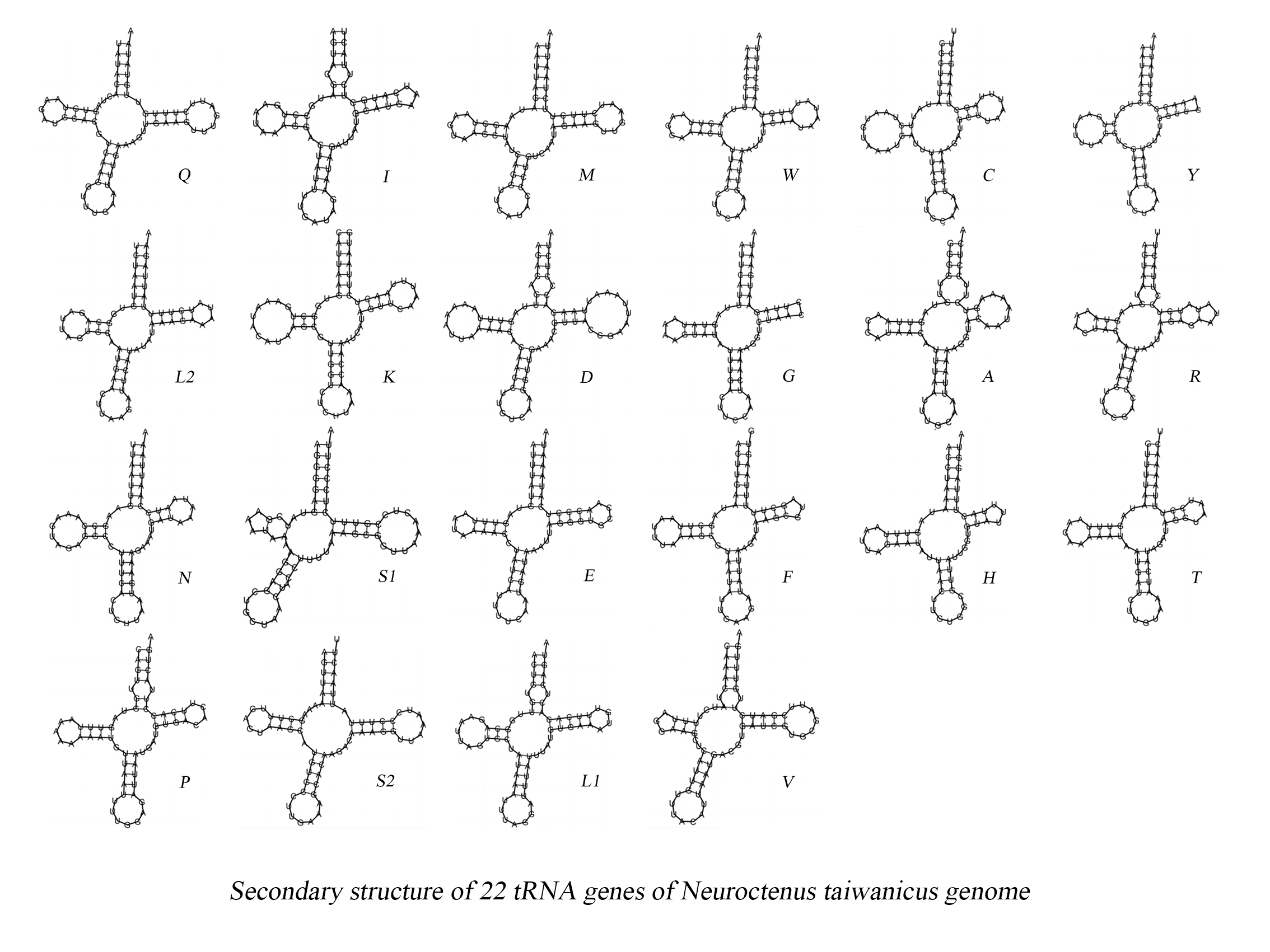

Supplement: Supplemental Material [file TMDN_A_2335986_SM3645.png]
